# Interpregnancy interval and adverse birth outcomes: a population-based cohort study of twins

**DOI:** 10.1186/s12884-023-06119-x

**Published:** 2024-01-31

**Authors:** Gursimran Dhamrait, Melissa O’Donnell, Hayley Christian, Catherine L. Taylor, Gavin Pereira

**Affiliations:** 1 Telethon Kids Institute, The University of Western Australia, 15 Hospital Avenue, PO Box 855, West Perth, Nedlands, Western Australia 6872 Australia; 2 School of Population and Global Health, The University of Western Australia, Nedlands, Western Australia Australia; 3 Australian Centre for Child Protection, University of South Australia, Adelaide, South Australia Australia; 4 Centre for Child Health Research, The University of Western Australia, Nedlands, Western Australia Australia; 5 Curtin School of Population Health, Curtin University, Perth, Australia; 6 Centre for Fertility and Health (CeFH), Norwegian Institute of Public Health, Oslo, Norway; 7 enAble Institute, Curtin University, Perth, Western Australia Australia

**Keywords:** Interpregnancy intervals, Low birth weight, Preterm birth, Early preterm birth, Small for gestational age, Record linkage, Twins, Australia

## Abstract

**Background:**

To investigate associations between interpregnancy intervals (IPIs) and adverse birth outcomes in twin pregnancies.

**Methods:**

This retrospective cohort study of 9,867 twin pregnancies in Western Australia from 1980–2015. Relative Risks (RRs) were estimated for the interval prior to the pregnancy (IPI) as the exposure and after the pregnancy as a negative control exposure for preterm birth (< 37 weeks), early preterm birth (< 34 weeks), small for gestational age (SGA: < 10^th^ percentile of birth weight by sex and gestational age) and low birth weight (LBW: birthweight < 2,500 g).

**Results:**

Relative to IPIs of 18–23 months, IPIs of < 6 months were associated with a higher risk of early preterm birth (aRR 1.41, 95% CI 1.08–1.83) and LBW for at least one twin (aRR 1.16, 95% CI 1.06–1.28). IPIs of 6–11 months were associated with a higher risk of SGA (aRR 1.24, 95% CI 1.01–1.54) and LBW for at least one twin (aRR 1.09, 95% CI 1.01–1.19). IPIs of 60–119 months and ≥ 120 months were associated with an increased risk of preterm birth (RR 1.12, 95% CI 1.03–1.22; and (aRR 1.25, 95% CI 1.10–1.41, respectively), and LBW for at least one twin (aRR 1.17, 95% CI 1.08–1.28; and aRR 1.20, 95% CI 1.05–1.36, respectively). IPIs of ≥ 120 months were also associated with an increased risk of early preterm birth (aRR 1.42, 95% CI 1.01–2.00). After negative control analysis, IPIs ≥ 120 months remained associated with early preterm birth and LBW.

**Conclusion:**

Evidence for adverse associations with twin birth outcomes was strongest for long IPIs.

**Supplementary Information:**

The online version contains supplementary material available at 10.1186/s12884-023-06119-x.

## Introduction

The prevalence rates of multifetal pregnancies and, therefore, of multiple births have increased substantially worldwide [1]. These increases have been primarily attributed to the increased use of assisted reproductive technologies and advanced maternal age at conception [1]. Compared to singleton pregnancies, multifetal pregnancies are associated with higher rates of pregnancy complications and adverse neonatal and perinatal outcomes, irrespective of conception method [2–4]. Twins are more likely to be born preterm [5, 6] and be classified as low birth weight (LBW) [5, 7]. However, there is a paucity of research investigating the effects of modifiable risk factors such as birth spacing on adverse birth outcomes in twin pregnancies.

The time between pregnancies, including the birth-to-birth interval and interpregnancy interval (IPI), is a modifiable risk factor that has the potential to reduce the risk of adverse birth outcomes such as preterm birth, small for gestational age at birth (SGA) and LBW [8]. The World Health Organization (WHO) recommends an IPI of at least 24 months to reduce the risk of adverse perinatal outcomes [9]. These recommendations are based primarily on singleton studies that have reported a strong U-shaped relationship between various adverse birth outcomes and IPIs, whereby both short (< 6 months) and long (> 60 months) IPIs are associated with an increased risk, whilst IPIs of 18–23 months have been reported to be associated with the lowest risk of adverse birth outcomes [8, 10]. Given the elevated risk of adverse birth outcomes in twin pregnancies, the optimal duration of IPIs and risks associated with sub-optimal IPIs, are likely to differ from those observed in singleton pregnancies. The two studies to date that examined the association between IPIs and adverse birth outcomes in twin pregnancies, have reported conflicting findings [11, 12].

Singleton studies have proposed several theories to explain the possible biological pathways between IPIs and adverse child outcomes. The two primary hypotheses in support of IPIs having a causal role are the *maternal depletion hypothesis*,^13^ and the *physiological regression hypothesis* [14]. The *maternal depletion hypothesis* suggests that short IPIs leave mothers with insufficient recovery time from the physiological stresses of a previous pregnancy and subsequent lactation [13]. Twin pregnancies are typically more depleting in comparison to singleton pregnancies, and thus, avoiding shorter IPIs may be even more beneficial after multifetal pregnancies to allow for sufficient maternal recovery time. Alternatively, the *physiological regression hypothesis* proposes that maternal physiological processes are primed for fetal growth during pregnancy and gradually decline over time post-delivery. Thus, long IPIs are proposed to result in the loss of the benefits in terms of physiological adaptation from the previous pregnancy resulting in a state resembling a primigravida [15]. Furthermore, the effects of long IPIs may be further compounded by advanced maternal age [16].

An alternative hypothesis is that the associations between IPIs and adverse pregnancy outcomes can be explained partially by systematic bias [17]. The elevated risk of suboptimal IPIs may be associated with other factors, such as socioeconomic status, breastfeeding and other antenatal and postnatal practices, that are causally associated with adverse birth outcomes [18]. Singleton studies via a negative-control analysis using the post-birth IPI (defined as the time interval between the birth of a child and start of the pregnancy of their next youngest sibling) to predict the outcomes of the prior-born sibling provide evidence for the *systematic bias hypothesis* [19].

This study examined the associations between IPIs leading to twin pregnancies and adverse birth outcomes (preterm birth, early preterm birth, SGA, and LBW) and determined if these associations can be observed after adjustment for post-birth IPI, a negative control exposure.

## Methods

In this retrospective population-based cohort study, we obtained anonymised individual-level perinatal and birth-related data from the Midwives Notification System and the Birth Registry. The Midwives Notification System is a statutory collection of all births (still- or live-born) in Western Australia (WA), with either a birthweight > 400 g and/or a final gestational length ≥ 20 weeks. Data for inclusion in this database are recorded for all births by the attending midwife in hospitals or at home in WA. We then selected mothers who delivered twins between the period of 1 January 1980 to 31 December 2015 and who had either; 1) prior birth enabling IPI derivation; and/or 2) subsequent birth enabling post-birth IPI derivation. Data linkage was conducted by the WA Data Linkage Branch from the Department of Health WA [20].

### Study population

**Fig. 1:**
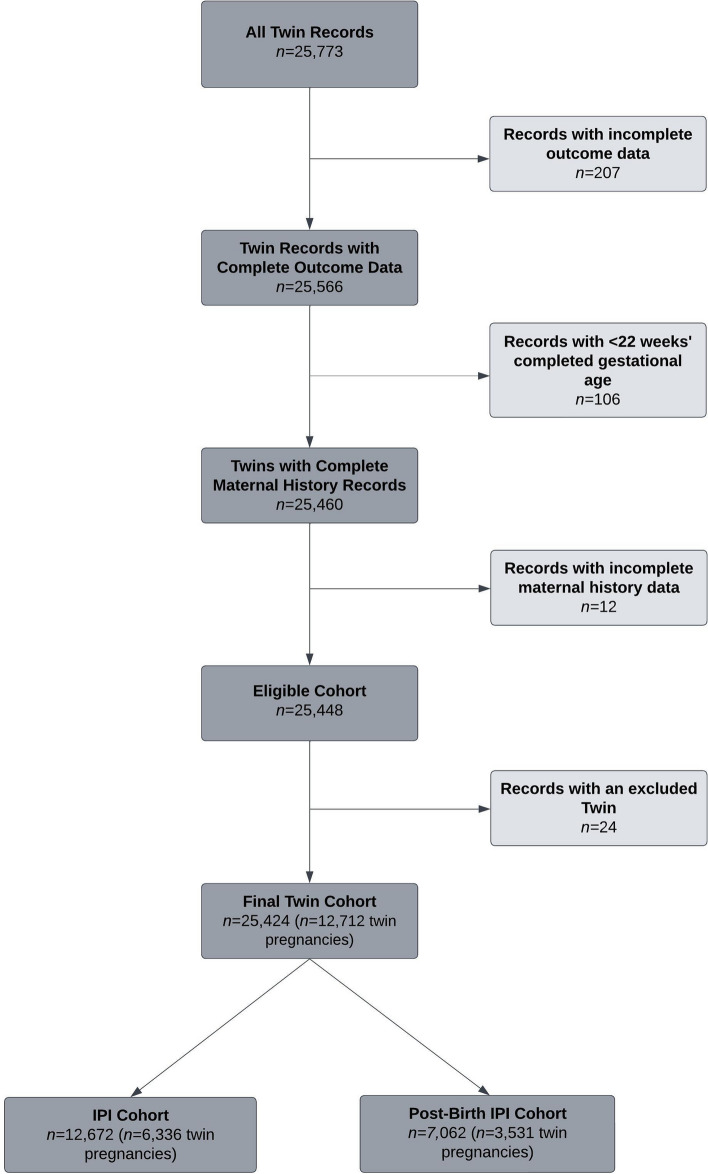
Eligible Cohort and Numbers Included for Analyses

The study population included all twin children born in WA during the study period (*n* = 25,773; Fig. 1). Records for twins were sequentially excluded if they had: 1) incomplete data for SGA classifications (*n* = 207); 2) missing gestational age or < 22 weeks of completed gestation (*n* = 106); 3) missing previous maternal history of adverse birth outcomes data (*n* = 12), and 4) a co-twin who was excluded based on either of the two previous exclusion criteria (*n* = 24). After these exclusions, 25,424 children remained (*n* = 12,712 twin pregnancies). Records with < 22 weeks of completed gestation were excluded because of a high proportion of these records being classified low birth weight and small for gestational age, and thus have the potential to bias adverse birth outcome statistics. To form the IPI cohort, pregnancies were further sequentially excluded if they had: 1) a parity of 0, i.e., twins were firstborns (*n* = 5,115); and 2) missing IPIs (*n* = 1,261). Overall, this study examined a total of 9,867 pregnancies, with the IPI cohort comprised of 6,336 pregnancies (*n* = 12,672 twins), and a total of 3,531 pregnancies (*n* = 7,602 twins) comprised the post-birth IPI cohort (negative control exposure).

### Exposure variables

#### Interpregnancy intervals

IPI was derived as the time from the birth of the immediately older sibling to the start of the twin pregnancy (Supplementary Fig. 1). The start of the twin pregnancy was derived as the birth date minus the gestational age at birth, measured in completed weeks of gestation. In line with previous studies [8, 21–24], short IPIs were classified as; < 6, 6–11, and 12–17 months, and long IPIs were classified as; 24–59, 60–119, and ≥ 120 months. The reference category was an IPI of 18–23 months.

#### Post-birth interpregnancy intervals

The post-birth IPI was derived as the time between the end of the twin pregnancy (i.e., birth of the twins) and the start of the immediate subsequent pregnancy (Supplementary Fig. 1). Post-birth IPI was used as a negative exposure under the assumption that in the absence of confounding factors: 1) post-birth IPI cannot *directly* affect the birth outcomes of twins in the previous pregnancy; and 2) any *reverse causation* effects of the post-birth IPI on the prior twin pregnancy, would be negligible [19].

### Outcome measures

Outcome variables were preterm birth (gestational age < 37 completed weeks), early preterm birth (gestational age < 34 completed weeks), LBW (birthweight < 2,500 g), and SGA (< 10^th^ centile of Australian national birthweight centiles by sex and gestational age in weeks). In the IPI cohort the prevalence for one twin and both twins being classified as SGA was 13.2% and 2.3% respectively, whilst prevalence for one twin and both twins being classified as LBW was 23.1% and 33.6% respectively. The prevalence of one twin and both twins being classified as SGA was 19.6% and 3.7% respectively, whilst prevalence for one twin and both twins being classified as LBW 22.1%, and 46.5% respectively, for all pregnancies with an available post-birth IPI. Thus, variables were derived to assess whether at least one twin per pregnancy was classified as either LBW or SGA.

### Adjustment variables

All adjustment variables were identified at the time of the birth of the twin pregnancy for both the IPI and post-birth IPI cohorts. We adjusted for maternal age (categorical variable: < 25; 25–29; 30–34; and ≥ 35 years), marital status, parity, birth year and socioeconomic status, and maternal ethnicity (categorised as either: Caucasian; Aboriginal/Torres Strait Islander; or All Other) [8, 25]. We also adjusted for maternal occupational status at birth. Maternal occupation at birth was obtained from Birth Registrations and was coded as a four-digit standard code in line with the Australian and New Zealand Standard Classification of Occupations [26]. These codes were assigned a value ranging from 0–100 using the Australian Socioeconomic Index 2006 (AUSEI06) and then categorised into five groups; [0,20], (20,40], (40,60], (60,80] and (80,100] [27]. Low AUSEI06 values represented low-status occupations. Socioeconomic status was also defined using the Index of Relative Socioeconomic Disadvantage (IRSD), [28] which assigns geographical areas with a score from 1 (most disadvantaged) to 5 (least disadvantaged), using residential address at the time of the child’s birth was obtained from Birth Registrations. We adjusted for either the presence or absence of any adverse birth outcomes in any previous pregnancies for each mother per outcome variable, with respect to the twin pregnancy for both the IPI and post-birth IPI cohorts.

### Multiple imputation

Complete covariate information was available for 85.8% (*n* = 5,438) of pregnancies in the IPI cohort and 77.1% (*n* = 2,722) of pregnancies in the cohort used to examine the effects of post-birth IPI. A total of three covariates had missing data; i) maternal marital status at birth, ii) maternal occupation status scale, and vi) IRSD. Multiple imputation via chained equations, [29] using 20 imputed datasets, was applied to minimise bias attributable to missing data.

### Statistical modelling

The association between IPIs and the risk of twins being classified as either preterm or early preterm, low birth weight and SGA was modelled using modified Poisson regression with robust error variance to estimate the relative risk [30, 31]. Adjustment variables were added simultaneously to the models. Relative Risk (RR) and the associated 95% confidence intervals (CIs) were estimated for both exposure (IPI) and adjustment variables. Post-birth IPI (negative-control exposure) was used to estimate effects attributable to a predisposition to adverse birth outcomes and certain IPIs. The Ratio of Relative Risk (RoR) were derived as the adjusted RR for the association with IPI divided by the adjusted RR for the association with the negative control exposure. Therefore, an RoR of > 1 indicated that the association between adverse birth outcomes and IPI was greater than expected. All statistical analyses were conducted in SAS version 9.4 [32] (using SAS PROC GENMOD) [31].

### Sensitivity analysis

As a sensitivity analysis to assess the effect of multiple imputation, we compared the outcomes based on the imputed data to the complete cases only for i) the IPI cohort (Supplementary Table 1) and ii) for the post-birth IPI cohort (Supplementary Table 2). To assess the temporal effects of socioeconomic status (maternal occupational status scale and IRSD category) on the associations between interpregnancy intervals and adverse birth outcomes, we compared results from the imputed dataset adjusted for socioeconomic status at the time of the previous pregnancy compared to the imputed dataset adjusted for socioeconomic status at the time of the twin pregnancy to estimate (Supplementary Table 3).

## Results

The mean IPI was 32.7 months (standard deviation [SD]: 30.4), and the mean post-birth IPI was 34.4 months (SD: 30.5). Tables 1 and 2 show the sociodemographic characteristics of the study population. The prevalence of short (< 12 months) and long (≥ 60 months) IPIs was 17% and 13%, respectively. For all pregnancies included in the study population, the prevalence of preterm birth and early preterm birth was 53% and 15%, respectively (Supplementary Table 1). The prevalence of at least one twin classified as SGA and LBW was 16% and 57%, respectively (Supplementary Table 1). In the post-birth IPI cohort, the prevalence of preterm birth and early preterm birth was 60% and 24%, respectively (Supplementary Table 2). The prevalence of at least one twin classified as SGA and LBW was 23% and 69%, respectively, for all pregnancies with an available post-birth IPI (Supplementary Table 2).

**Table 1:** Characteristics of twin pregnancies analysed in the IPI cohort

**Characteristic**	**Total**	**Interpregnancy Interval** *n* (%)						
		< 6	6- 11	12–17	18–23	24–59	60–119	≥ 120
	*n* = 6336	293 (4.6)	797 (12.6)	1106 (17.5)	1017 (16.1)	2300 (36.3)	652 (10.3)	171 (2.7)
**Maternal History of Preterm Birth**								
No^a^	5564 (87.8)	207 (70.6)	685 (86.1)	985 (89.1)	909 (89.4)	2045 (88.9)	589 (90.3)	144 (84.2)
Yes	772 (12.2)	86 (29.4)	112 (14.1)	121 (10.9)	108 (10.6)	255 (11.1)	63 (9.7)	27 (15.8)
**Maternal History of Early Preterm Birth**								
No^a^	6066 (95.7)	240 (81.9)	757 (95.1)	1066 (96.4)	979 (96.3)	2227 (96.8)	634 (97.2)	163 (95.3)
Yes	270 (4.3)	53 (18.1)	40 (5.0)	40 (3.6)	38 (3.7)	73 (3.2)	18 (2.8)	8 (4.7)
**Maternal History of Small for Gestational age**								
No^a^	5444 (85.9)	216 (73.7)	681 (85.6)	969 (87.6)	889 (87.4)	1997 (86.8)	550 (84.4)	142 (83.0)
Yes	892 (14.1)	77 (26.3)	116 (14.6)	137 (12.4)	128 (12.6)	303 (13.2)	102 (15.6)	29 (17.0)
**Maternal History of Low Birth Weight**								
No^a^	5712 (90.2)	210 (71.7)	710 (89.2)	1010 (91.3)	935 (91.9)	2099 (91.3)	601 (92.2)	147 (86.0)
Yes	624 (9.8)	83 (28.3)	87 (10.9)	96 (8.7)	82 (8.1)	201 (8.7)	51 (7.8)	24 (14.0)
**Parity**								
1^a^	3553 (56.1)	126 (43)	445 (55.9)	690 (62.4)	639 (62.8)	1279 (55.6)	292 (44.8)	82 (48.0)
2	1679 (26.5)	75 (25.6)	193 (24.2)	260 (23.5)	247 (24.3)	642 (27.9)	215 (33.0)	47 (27.5)
≥ 3	1104 (17.4)	92 (31.4)	159 (20.0)	156 (14.1)	131 (12.9)	379 (16.5)	145 (22.2)	42 (24.6)
**Maternal Marital Status**								
Married^a^	5775 (91.1)	251 (85.7)	718 (90.2)	1042 (94.2)	960 (94.4)	2107 (91.6)	561 (86.0)	136 (79.5)
Other	541 (8.5)	41 (14.0)	74 (9.3)	60 (5.4)	53 (5.2)	190 (8.3)	89 (13.7)	34 (19.9)
Missing	20 (0.3)	< 5	5 (0.6)	< 5	< 5	< 5	< 5	< 5
**Maternal Ethnicity**								
Caucasian^a^	5405 (85.3)	222 (75.8)	667 (83.8)	977 (88.3)	899 (88.4)	1946 (84.6)	546 (83.7)	148 (86.5)
Indigenous Australian	452 (7.1)	43 (14.7)	66 (8.3)	57 (5.2)	53 (5.2)	165 (7.2)	53 (8.1)	15 (8.8)
All Other	479 (7.6)	28 (9.6)	64 (8.0)	72 (6.5)	65 (6.4)	189 (8.2)	53 (8.1)	8 (4.7)
**Maternal Age (years) at Twins’ Birth**								
< 24	788 (12.4)	83 (28.3)	149 (18.7)	161 (14.6)	117 (11.5)	258 (11.2)	20 (3.1)	0 (0)
25-29^a^	1869 (29.5)	94 (32.1)	298 (37.4)	316 (28.6)	348 (34.2)	637 (27.7)	168 (25.8)	8 (4.7)
30–34	2193 (34.6)	72 (24.6)	221 (27.8)	411 (37.2)	351 (34.5)	825 (35.9)	259 (39.7)	54 (31.6)
≥ 35	1486 (23.5)	44 (15.0)	129 (16.2)	218 (19.7)	201 (19.8)	580 (25.2)	205 (31.4)	109 (63.7)
**Maternal Occupation Status Scale**^b^** (quintiles)**								
0–20	2279 (36.0)	115 (39.2)	284 (35.7)	372 (33.6)	364 (35.8)	870 (37.8)	240 (36.8)	34 (19.9)
> 20–40	1105 (17.4)	60 (20.5)	124 (15.6)	158 (14.3)	157 (15.4)	396 (17.2)	155 (23.8)	55 (32.2)
> 40–60	1016 (16.0)	23 (7.8)	121 (15.2)	191 (17.3)	163 (16.0)	372 (16.2)	105 (16.1)	41 (24.0)
> 60–80	422 (6.7)	13 (4.4)	40 (5.0)	84 (7.6)	72 (7.1)	151 (6.6)	47 (7.2)	15 (8.8)
> 80-100^a^	815 (12.9)	30 (10.2)	90 (11.3)	174 (15.7)	142 (14.0)	299 (13.0)	67 (10.3)	13 (7.6)
Missing	699 (11.0)	52 (17.7)	138 (17.3)	127 (11.5)	119 (11.7)	212 (9.2)	38 (5.8)	13 (7.6)
**Birth Year**								
1980-1989^a^	1013 (16.0)	65 (22.2)	168 (21.1)	199 (18.0)	170 (16.7)	371 (16.1)	40 (6.1)	0 (0)
1990–1999	1818 (28.7)	74 (25.3)	211 (26.5)	306 (27.7)	312 (30.7)	664 (28.9)	213 (32.7)	38 (22.2)
2000–2009	2125 (33.5)	92 (31.4)	248 (31.2)	362 (32.7)	315 (31.0)	756 (32.9)	261 (40.0)	91 (53.2)
2010–2015	1380 (21.8)	62 (21.2)	170 (21.4)	239 (21.6)	220 (21.6)	509 (22.1)	138 (21.2)	42 (24.6)
**Index of Relative Socioeconomic Disadvantage**^c^** (quintiles)**								
1 (most disadvantaged)	1242 (19.6)	103 (35.2)	152 (19.1)	182 (16.5)	172 (16.9)	452 (19.7)	140 (21.5)	41 (24.0)
2	1231 (19.4)	68 (23.2)	170 (21.4)	196 (17.7)	171 (16.8)	437 (19.0)	156 (23.9)	33 (19.3)
3	1130 (17.8)	52 (17.7)	145 (18.2)	201 (18.2)	178 (17.5)	392 (17.0)	121 (18.6)	41 (24.0)
4	1238 (19.5)	32 (10.9)	157 (19.7)	231 (20.9)	216 (21.2)	461 (20.0)	112 (17.2)	29 (17.0)
5 (least disadvantaged)^a^	1268 (20.0)	28 (9.6)	139 (17.5)	244 (22.1)	238 (23.4)	485 (21.1)	108 (16.6)	26 (15.2)
Missing	227 (3.6)	10 (3.4)	34 (4.3)	52 (4.7)	42 (4.1)	73 (3.2)	15 (2.3)	< 5

^a^Reference group for regression analysis

^b^Maternal and Paternal Occupation Status are classified into five categories in line with the Australian Socioeconomic Index 2006 (AUSEI06); low AUSEI06 values represent low-status occupations

^c^Categorised as nationally defined quintiles (1 = most disadvantaged to 5 = least disadvantaged); as quintiles are defined nationally (rather than within study population), numbers within each category vary from 20% of the total

**Table 2:** Characteristics of twin pregnancies analysed in the Post-Birth IPI cohort

**Characteristic**	**Total**	**Post-Birth Interpregnancy Interval** *n* (%)						
		< 6	6–11	12–17	18–23	24–59	60–119	≥ 120
	*n* = 3531	223 (6.3)	434 (12.3)	533 (15.1)	413 (11.7)	1359 (38.5)	477 (13.5)	92 (2.6)
**Maternal History of Preterm Birth**								
No^a^	3353 (95.0)	207 (92.8)	406 (93.5)	503 (94.4)	394 (95.4)	1296 (95.4)	457 (95.8)	90 (97.8)
Yes	178 (5.0)	16 (7.2)	28 (6.5)	30 (5.6)	19 (4.6)	63 (4.6)	20 (4.2)	< 5
**Maternal History of Early Preterm Birth**								
No^a^	3464 (98.1)	219 (98.2)	418 (96.3)	524 (98.3)	404 (97.8)	1338 (98.5)	469 (98.3)	92 (100)
Yes	67 (1.9)	< 5	16 (3.7)	9 (1.7)	9 (2.2)	21 (1.5)	8 (1.7)	0 (0)
**Maternal History of Small for Gestational age**								
No^a^	3305 (93.6)	205 (91.9)	397 (91.5)	498 (93.4)	384 (93.0)	1291 (95.0)	442 (92.7)	88 (95.7)
Yes	226 (6.4)	18 (8.1)	37 (8.5)	35 (6.6)	29 (7.0)	68 (5.0)	35 (7.3)	< 5
**Maternal History of Low Birth Weight**								
No^a^	3374 (95.6)	211 (94.6)	398 (91.7)	509 (95.5)	395 (95.6)	1310 (96.4)	462 (96.9)	89 (96.7)
Yes	157 (4.4)	12 (5.4)	36 (8.3)	24 (4.5)	18 (4.4)	49 (3.6)	15 (3.1)	< 5
**Parity**								
0	1999 (56.6)	100 (44.8)	211 (48.6)	288 (54.0)	251 (60.8)	834 (61.4)	269 (56.4)	46 (50.0)
1^a^	883 (25.0)	59 (26.5)	119 (27.4)	142 (26.6)	99 (24.0)	308 (22.7)	128 (26.8)	28 (30.4)
2	363 (10.3)	34 (15.2)	53 (12.2)	52 (9.8)	32 (7.7)	133 (9.8)	49 (10.3)	10 (10.9)
≥ 3	286 (8.1)	30 (13.5)	51 (11.8)	51 (9.6)	31 (7.5)	84 (6.2)	31 (6.5)	8 (8.7)
**Maternal Marital Status**								
Married^a^	3072 (87)	195 (87.4)	386 (88.9)	477 (89.5)	358 (86.7)	1192 (87.7)	392 (82.2)	72 (78.3)
Other	447 (12.7)	28 (12.6)	47 (10.8)	54 (10.1)	54 (13.1)	164 (12.1)	80 (16.8)	20 (21.7)
Missing	12 (0.3)	0 (0)	< 5	< 5	< 5	< 5	5 (1.0)	0 (0)
**Maternal Ethnicity**								
Caucasian^a^	2962 (83.9)	178 (79.8)	349 (80.4)	440 (82.6)	353 (85.5)	1151 (84.7)	414 (86.8)	77 (83.7)
Indigenous Australian	292 (8.3)	23 (10.3)	40 (9.2)	47 (8.8)	34 (8.2)	114 (8.4)	27 (5.7)	7 (7.6)
All Other	277 (7.8)	22 (9.9)	45 (10.4)	46 (8.6)	26 (6.3)	94 (6.9)	36 (7.5)	8 (8.7)
**Maternal Age (years) at Twins’ Birth**								
< 24	1164 (33.0)	66 (29.6)	104 (24.0)	147 (27.6)	132 (32.0)	452 (33.3)	207 (43.4)	56 (60.9)
25-29^a^	1273 (36.1)	75 (33.6)	154 (35.5)	170 (31.9)	147 (35.6)	509 (37.5)	185 (38.8)	33 (35.9)
30–34	838 (23.7)	55 (24.7)	121 (27.9)	158 (29.6)	107 (25.9)	317 (23.3)	77 (16.1)	< 5
≥ 35	256 (7.3)	27 (12.1)	55 (12.7)	58 (10.9)	27 (6.5)	81 (6.0)	8 (1.7)	0 (0)
**Maternal Occupation Status Scale**^b^** (quintiles)**								
0–20	1016 (28.8)	75 (33.6)	129 (29.7)	140 (26.3)	115 (27.8)	370 (27.2)	152 (31.9)	35 (38.0)
> 20–40	633 (17.9)	43 (19.3)	70 (16.1)	96 (18.0)	67 (16.2)	248 (18.2)	93 (19.5)	16 (17.4)
> 40–60	551 (15.6)	28 (12.6)	74 (17.1)	91 (17.1)	71 (17.2)	206 (15.2)	69 (14.5)	12 (13.0)
> 60–80	181 (5.1)	10 (4.5)	28 (6.5)	28 (5.3)	23 (5.6)	69 (5.1)	22 (4.6)	< 5
> 80-100^a^	436 (12.3)	15 (6.7)	59 (13.6)	75 (14.1)	53 (12.8)	190 (14.0)	40 (8.4)	< 5
Missing	714 (20.2)	52 (23.3)	74 (17.1)	103 (19.3)	84 (20.3)	276 (20.3)	101 (21.2)	24 (26.1)
**Birth Year**								
1980-1989^a^	997 (28.2)	79 (35.4)	106 (24.4)	138 (25.9)	113 (27.4)	394 (29.0)	139 (29.1)	28 (30.4)
1990–1999	1084 (30.7)	73 (32.7)	132 (30.4)	151 (28.3)	120 (29.1)	399 (29.4)	161 (33.8)	48 (52.2)
2000–2009	1150 (32.6)	46 (20.6)	138 (31.8)	172 (32.3)	128 (31.0)	475 (35.0)	175 (36.7)	16 (17.4)
2010–2015	300 (8.5)	25 (11.2)	58 (13.4)	72 (13.5)	52 (12.6)	91 (6.7)	< 5	0 (0)
**Index of Relative Socioeconomic Disadvantage**^c^** (quintiles)**								
1 (most disadvantaged)	773 (21.9)	48 (21.5)	93 (21.4)	112 (21.0)	82 (19.9)	300 (22.1)	117 (24.5)	21 (22.8)
2	737 (20.9)	57 (25.6)	82 (18.9)	91 (17.1)	64 (15.5)	290 (21.3)	131 (27.5)	22 (23.9)
3	688 (19.5)	46 (20.6)	87 (20.0)	99 (18.6)	96 (23.2)	254 (18.7)	84 (17.6)	22 (23.9)
4	619 (17.5)	31 (13.9)	87 (20.0)	97 (18.2)	75 (18.2)	237 (17.4)	79 (16.6)	13 (14.1)
5 (least disadvantaged)^a^	577 (16.3)	30 (13.5)	70 (16.1)	107 (20.1)	74 (17.9)	223 (16.4)	62 (13.0)	11 (12.0)
Missing	137 (3.9)	11 (4.9)	15 (3.5)	27 (5.1)	22 (5.3)	55 (4.0)	< 5	< 5

^a^Reference group for regression analysis

^b^Maternal and Paternal Occupation Status are classified into five categories in line with the Australian Socioeconomic Index 2006 (AUSEI06); low AUSEI06 values represent low-status occupations

^c^Categorised as nationally defined quintiles (1 = most disadvantaged to 5 = least disadvantaged); as quintiles are defined nationally (rather than within study population), numbers within each category vary from 20% of the total

### Associations between IPIs and adverse birth outcomes

**Fig. 2:**
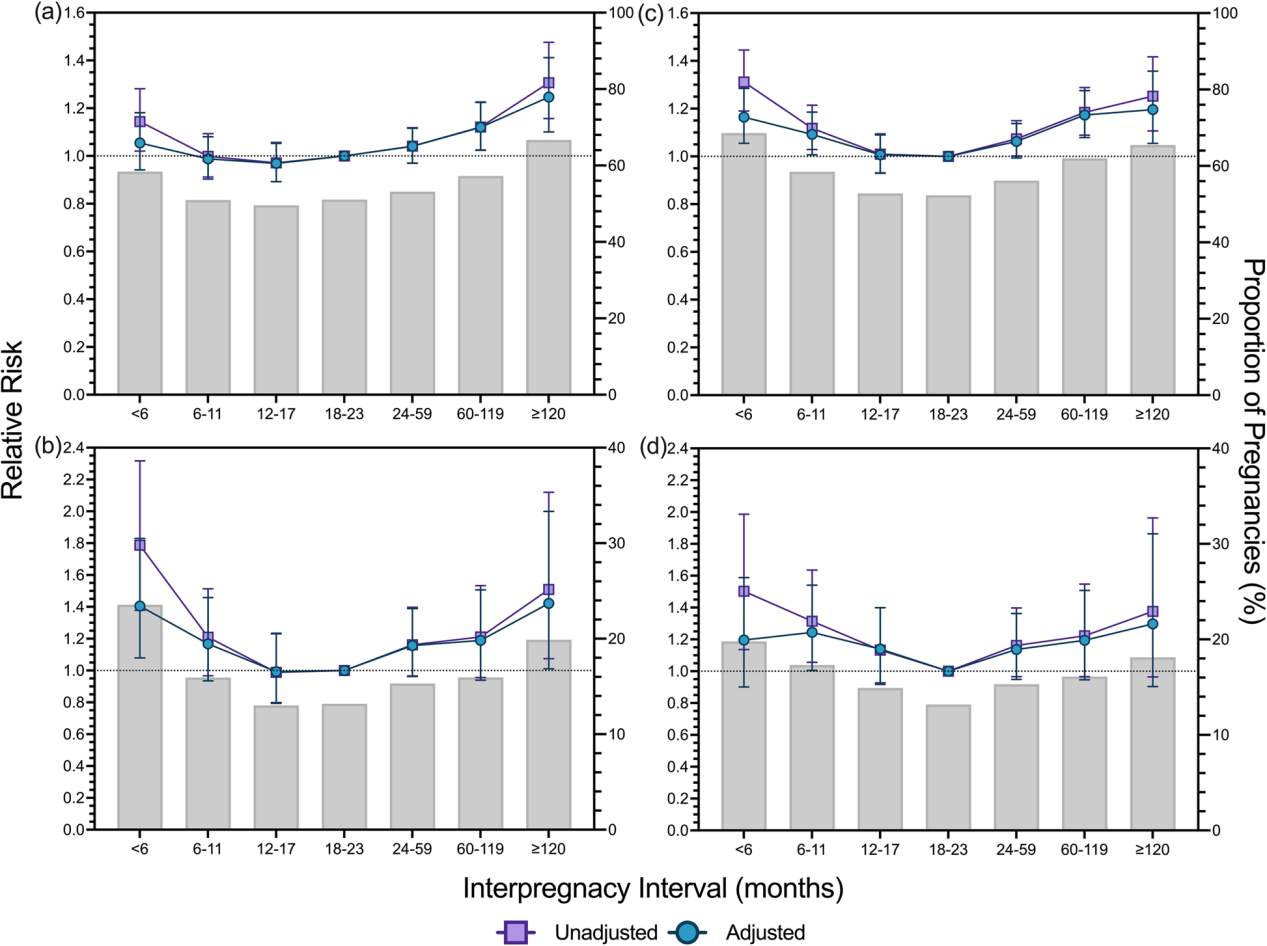
Unadjusted and adjusted Relative Risk from interaction models for the association between interpregnancy intervals and adverse birth outcomes in twin pregnancies. The prevalence rate of adverse birth outcomes: (**a**) preterm birth; (**b**) early preterm birth; (**c**) at least one twin being classified as low birth weight; and (**d**) at least one twin being classified as small for gestational age, is overlayed with the relative risk of adverse birth outcomes for each outcome. Adjusted model based on pooled analysis from 20 imputed datasets controlling for: parity, birth year category, maternal ethnicity, maternal marital status at time of birth, maternal age at time of birth, maternal occupational status scale at time of birth, previous maternal history for each respective outcome variable, and Index of Relative Socioeconomic Disadvantage category. All relative risk data is presented with 95% confidence intervals: modified Poisson Regression

Both unadjusted and adjusted IPIs exhibited U-shaped associations with adverse birth outcomes (Fig. 2). In the adjusted models, short IPIs of < 6 months were associated with a higher risk of early preterm birth (aRR 1.41, 95% CI 1.08–1.83) and at least one twin being classified as LBW (aRR 1.16, 95% CI 1.06–1.28), only (Table 3). IPIs of 6–11 months were associated with a higher risk of at least one twin classified being as SGA (aRR 1.25, 95% CI 1.01–1.54) and LBW (aRR 1.09, 95% CI 1.01–1.18). Longer IPIs of 60–119 months were associated with a higher risk of preterm birth (aRR 1.12, 95% CI 1.03–1.22) and LBW (aRR 1.17, 95% CI 1.08–1.28). Very long IPIs of ≥ 120 months were associated with increased risk of preterm birth (aRR 1.25, 95% CI 1.10–1.41), early preterm birth (aRR 1.42, 95% CI 1.01–2.00) and LBW (aRR 1.20, 95% CI 1.05–1.36).

**Table 3:** Relative Risk (RR)^1^ and Ratio of Ratios (RoR)^2^ for the association between; preterm birth, early preterm birth, and at least one twin being classified as low birth weight or small for gestational age and interpregnancy intervals (IPIs) in twin pregnancies

**Outcome Variable and Cohort**		**Interpregnancy Interval** (months)						
		aRR [95% CI]^c^						
		< 6	6–11	12–17	18–23	24–59	60–119	≥ 120
**Preterm Birth**	IPI^a^	1.05 [0.94–1.18]	0.99 [0.90–1.08]	0.97 [0.89–1.05]	1 [referent]	1.04 [0.97–1.12]	1.12 [1.02–1.22]^**^	1.25 [1.10–1.41]^***^
	Post-Birth IPI^a^	1.37 [1.22–1.53]^***^	1.22 [1.10–1.36]^***^	1.08 [0.97–1.20]	1 [referent]	1.01 [0.92–1.11]	0.96 [0.85–1.08]	0.98 [0.80–1.21]
	RoR^b^	**0.77 [0.66–0.91]**	**0.81 [0.70–0.93]**	0.90 [0.79–1.03]	1 [referent]	1.03 [0.92–1.16]	**1.17 [1.01–1.35]**	1.27 [1.00–1.62]
**Early Preterm Birth**	IPI	1.41 [1.08–1.83]^*^	1.17 [0.94–1.46]	0.99 [0.80–1.23]	1 [referent]	1.16 [0.96–1.39]	1.19 [0.94–1.51]	1.42 [1.01–2.00]^*^
	Post-Birth IPI	2.03 [1.61–2.55]^***^	1.45 [1.16–1.81]^**^	0.99 [0.78–1.26]	1 [referent]	0.88 [0.72–1.07]	0.84 [0.66–1.08]	0.73 [0.45–1.19]
	RoR	**0.69 [0.49–0.99]**	0.81 [0.59–1.10]	1.00 [0.73–1.38]	1 [referent]	**1.32 [1.01–1.73]**	**1.41 [1.01–2.00]**	**1.94 [1.08–3.53]**
**Small for Gestational Age**	IPI	1.20 [0.90–1.59]	1.24 [1.01–1.54]^*^	1.14 [0.93–1.40]	1 [referent]	1.14 [0.95–1.36]	1.19 [0.95–1.51]	1.30 [0.90–1.86]
	Post-Birth IPI	1.50 [1.17–1.92]^***^	1.13 [0.90–1.42]	0.85 [0.67–1.08]	1 [referent]	0.86 [0.71–1.05]	0.90 [0.71–1.14]	0.93 [0.61–1.41]
	RoR	0.80 [0.55–1.16]	1.10 [0.80–1.50]	1.34 [0.98–1.83]	1 [referent]	**1.32 [1.01–1.72]**	1.33 [0.95–1.85]	1.39 [0.80–2.42]
**Low Birth Weight**	IPI	1.16 [1.06–1.28]^**^	1.09 [1.01–1.19]^*^	1.01 [0.93–1.09]	1 [referent]	1.06 [0.99–1.14]	1.17 [1.08–1.28]^***^	1.20 [1.05–1.36]^**^
	Post-Birth IPI	1.30 [1.19–1.42]^***^	1.17 [1.07–1.27]^***^	1.03 [0.94–1.12]	1 [referent]	1.01 [0.93–1.09]	0.99 [0.90–1.09]	0.87 [0.72–1.05]
	RoR	0.90 [0.78–1.02]	0.93 [0.83–1.05]	0.98 [0.87–1.11]	1 [referent]	1.05 [0.95–1.17]	**1.19 [1.05–1.35]**	**1.38 [1.10–1.73]**

IPI was defined as the time between the birth of twins (i.e., IPI cohort pregnancy) and the start of the subsequent pregnancy (*n* = 6,336 twin pregnancies)

Post-birth IPI was defined as the time between the birth of the twins (i.e., post-birth IPI cohort pregnancy) and the start of pregnancy of the immediately subsequent pregnancy (*n* = 3,531 twin pregnancies)

^a^Data is presented as relative risk [95% Confidence Intervals]

^b^RoR was derived as the adjusted RR for the association with IPI divided by the adjusted RR for the association with post-birth IPI. Data is presented as Ratio of Relative Risk [95% Confidence Intervals]

^c^All data was based on pooled analysis from 20 imputed datasets and adjusted for parity, birth year category, maternal ethnicity, maternal marital status at time of birth, maternal age at time of birth, maternal occupational status scale at time of birth, previous maternal history for each respective outcome variable, and IRSD category, with respect to the twin pregnancy for both the IPI and post-birth IPI cohorts

^***^p < 0.001, ^**^p < 0.01, ^*^p < 0.05

### Associations with the Post-birth IPI and RoR

**Fig. 3:**
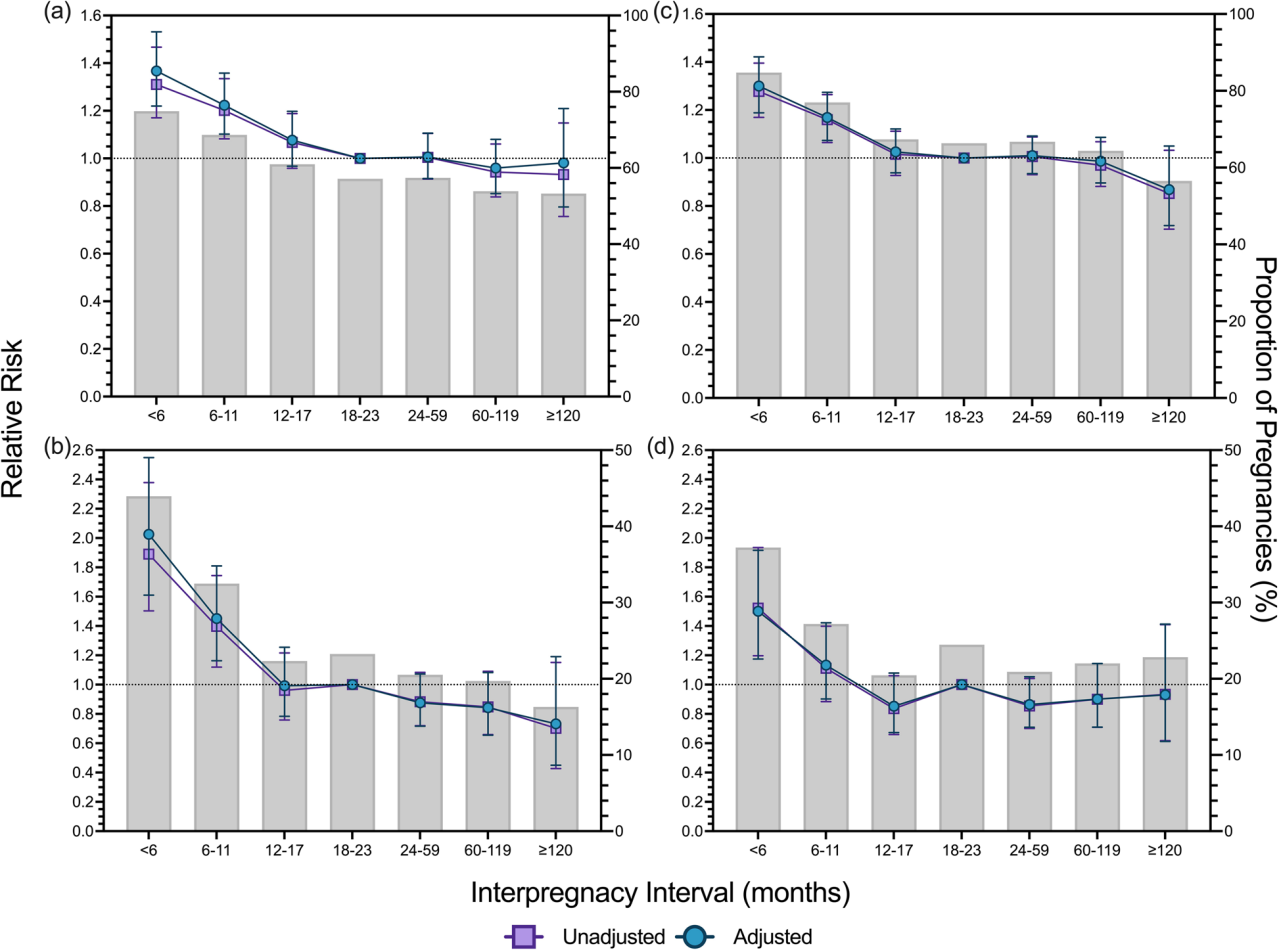
Unadjusted and adjusted Relative Risk from interaction models for the association between post-birth interpregnancy intervals and adverse birth outcomes in twin pregnancies. The prevalence rate of adverse birth outcomes: (**a**) preterm birth; (**b**) early preterm birth; (**c**) at least one twin being classified as low birth weight; and (**d**) at least one twin being classified as small for gestational age, is overlayed with the relative risk of adverse birth outcomes for each outcome. Adjusted model based on pooled analysis from 20 imputed datasets controlling for: parity, birth year category, maternal ethnicity, maternal marital status at time of birth, maternal age at time of birth, maternal occupational status scale at time of birth, previous maternal history for each respective outcome variable, and Index of Relative Socioeconomic Disadvantage category. All relative risk data is presented with 95% confidence intervals: modified Poisson Regression

In both unadjusted and adjusted models, short post-birth IPIs (< 6 months) were associated with a higher risk for all adverse birth outcomes (Fig. 3). Post-birth IPIs of 6–11 months were associated with higher risk of preterm birth (aRR 1.21, 95% CI 1.10–1.36), early preterm birth (aRR 1.45, 95% CI 1.16–1.81) and at least one twin being classified as LBW (aRR 1.17, 95% CI 1.07–1.27). There was insufficient evidence for associations between adverse birth outcomes and longer post-birth intervals (≥ 24 months). Longer post-birth IPIs were associated with a higher risk of preterm birth and early preterm birth than expected. However, for the IPI category of 24–59 months, IPIs had a larger effect than post-birth IPIs on the risk of at least one twin being classified as early preterm birth (RoR 1.32, 95% CI 1.01–1.73; Table 3) and SGA (RoR 1.32, 95% CI 1.01–1.72). Specifically, for the IPI category of 60–119 months, IPIs had a larger effect than post-birth IPIs on the risk of preterm birth (RoR 1.17, 95% CI 1.01–1.35), early preterm birth (RoR 1.41, 95% CI 1.01–2.00), and at least one twin being classified as LBW (RoR 1.19, 95% CI 1.05–1.35). Likewise, for the IPI category of ≥ 120 months, post-birth IPIs had a larger effect than expected on the risk of early preterm birth (RoR 1.94, 95% CI 1.08–3.53) and at least one twin being classified as LBW (RoR 1.38, 95% CI 1.10–1.73). However, shorter IPIs had a lower effect than post-birth IPIs on the risk of preterm birth and early preterm birth. Specifically, for the IPI category of < 6 months, IPIs had a lower effect than post-birth IPIs and were associated with a reduced risk of preterm birth (RoR 0.77, 95% CI 0.66–0.91) and early preterm birth (RoR 0.69, 95% CI 0.49–0.99). Additionally, for the IPI category of 6–11 months, IPIs had a lower effect than post-birth IPI with a reduced risk of preterm birth (RoR 0.81, 95% CI 0.70–0.93).

### Sensitivity analysis

The sensitivity analyses revealed that the overall associations between adverse birth outcomes and: i) IPIs (Supplementary Table 1); and ii) post-birth IPIs (Supplementary Table 2) were not substantially different between complete cases and the imputed cases.

## Discussion

### Main findings

Adverse birth outcomes for twin pregnancies were more prevalent when preceded by shorter (< 12 months) and longer IPIs (≥ 60 months) compared to those preceded by moderate IPIs (18–23 months) as recommended by the WHO [9]. Specifically, shorter IPIs were associated with increased risk of early preterm birth, SGA and LBW, and longer IPIs were significantly associated with preterm birth, early preterm birth, and LBW. Estimates of associations with longer IPIs were higher than expected (based on post-birth intervals) for each of the four birth outcomes examined. Overall, there is relatively stronger causal evidence for adverse birth outcomes from longer IPIs than shorter IPIs for this twin cohort.

### Strengths and limitations

The strengths of this study included the large cohort size, the use of population-based cohort design, the use of multiple IPI categories, the use of imputed data, control for maternal and socioeconomic variables, and the application of a negative control exposure. This study had several limitations. Firstly, for births in the period of 2010–2015, post-birth IPIs were limited to at most 95 months, due to the study end-date of 31 Dec 2017, thus limiting the population size and the inferences for post-birth IPIs RoRs corresponding to ≥ 120 months. Furthermore, we did not have information as to whether the twin pregnancies were planned and thus, are unable to ascertain differences in families who had children after a twin pregnancy, regardless of outcome and duration of post-birth IPI and those families that did not have children post twins. Although we expect only a small proportion of women would have received fertility treatment – approximately 3.6% of all women who give birth in Australia are estimated to undergo some form of assisted reproductive technology treatment [33] – we could not account for the use of assisted reproductive technologies. Administrative records do not include pregnancies ending before 20 weeks of gestations, we are unable to identify and account for miscarriages.

### Interpretation

We reported that 35% of women with twin pregnancies had short IPIs (< 18 months), and 13% of women had long IPIs (≥ 60 months). Although global estimates for the distribution of IPIs for singleton or twin pregnancies are not available, [34] two Australian singleton cohort studies reported that approximately 45% of women had IPIs of < 18 months, and 5–9% of women had IPIs of ≥ 60 months [8, 35]. An international cohort study using data from Australia, Finland, Norway and the USA reported that 6.7% and 12.4% of women had IPIs of < 6 months and ≥ 60 months, respectively [36]. Thus, it appears that short IPIs are less prevalent before twin births than singleton births, and long IPIs are more prevalent before twin births than singleton births. Furthermore, we observed that the mean IPI duration was shorter than the mean post-birth IPI duration, suggesting that, on average subsequent pregnancies after a twin pregnancy are delayed. In terms of the *maternal depletion hypothesis*, [13] it can be hypothesised that in comparison to singleton pregnancies, twin pregnancies and the subsequent lactation periods would be significantly more depleting and thus, delaying subsequent pregnancies after multifetal pregnancies to allow for sufficient maternal recovery time.

Our findings are in accordance with a population-based cohort study of 30,889 U.S women that reported that compared to IPIs of 18–36 months, short IPIs (4–17 months) were independently associated with an increased risk of several adverse neonatal outcomes, including preterm birth and extremely LBW [11]. We reported that short IPIs of both < 6 months and 6–11 months were independently associated with an increased risk of LBW, and IPIs of < 6 months were independently associated with an increased risk of early preterm birth. Combined, the findings of both studies add to the evidence base that the risk of adverse birth outcomes in twins may be associated with short IPIs. However, we reported that causal estimates of associations with shorter IPIs were lower than those expected for preterm and early preterm birth. This finding cannot be explained by reverse causation in the post-birth IPI analysis, as we would assume that parents would wait for a longer (not a shorter) period of time for the immediate sequelae of preterm and early preterm birth to resolve before conceiving another child for planned pregnancies. Moreover, although short IPIs and adverse birth outcomes might be associated with unplanned pregnancies, we are unaware of a reason why short intervals would be more likely to occur after adverse twin birth outcomes than before twin birth.

A Californian (USA) study of 189,931 s births, of which 1.2% were twin pregnancies, reported a significant interaction between IPI duration and twin pregnancy [12]. However, this study reported that compared to women with IPIs of > 18 months, women with short IPIs (< 6 and 6–18 months) had a decreased odds of preterm birth in twins [12]. Differences in findings across studies may be attributed to differences in the definition of the reference category used. Furthermore, the Californian study only included mothers who had a subsequent pregnancy within six years; thus, the maximum IPI duration for this study was 72 months. Given the inconsistent findings and reference categories used in existing studies, further research is required to better elucidate the association between short IPIs and adverse birth outcomes in twin pregnancies. Regardless of causality, clinically, short IPIs should be considered a useful marker of increased risk of adverse birth outcomes in twin pregnancies.

Our findings also showed a trend towards an increased risk of early preterm birth associated with longer IPIs (≥ 24 months), with the RoR significant for all long IPI categories (≥ 24 months). Similarly, we reported that longer IPIs (≥ 60 months) were independently associated with an increased risk of preterm birth and LBW, with the RoR being significant for the same IPI parameters. The US study also reported that compared to IPIs of 18–36 months, longer IPIs (≥ 61 months) were independently associated with a greater risk of preterm birth but not extremely LBW (< 1000 g) [11]. Although we reported similar results, the results of our study and the US study are not directly comparable as the US study included mothers of twins with only one prior birth and used generalised estimating equation models to account for twin clustering. Combined, the findings from the US study and our study add to the evidence base that the risk of adverse birth outcomes in twins may be causally associated with longer IPIs – thus, providing further evidence for the *physiological regression hypothesis*. In addition, women who conceive twin pregnancies soon after a first birth (i.e., have short IPIs) may be different from those who wait for longer intervals, even after adjustment for advanced maternal age and other socioeconomic factors. For example, pregnancies with longer IPIs may suffer from reduced fecundability; [37] therefore, associations between long IPIs and adverse birth outcomes in twins may be further compounded by the use of assisted reproductive technologies [38, 39]. Furthermore, it is likely that the relationship between IPIs and adverse birth outcomes in twins will vary across populations. As present research is limited to developed countries, future studies should also aim to assess the associations between IPIs and birth outcomes in twins in developing countries. Clinically, therefore closer monitoring for twin pregnancies with long IPIs may be required to reduce the risk of adverse birth outcomes, especially in twin pregnancies complicated by pregnancy and sociodemographic factors.

## Conclusion

IPIs exhibited independent U-shaped associations with adverse birth outcomes in twin pregnancies before accounting for post-birth IPIs (negative-control exposure). After accounting for negative-control exposure, IPIs of ten years or longer remained associated with increased risk of preterm birth, early preterm birth, and LBW. Therefore, evidence for associations with adverse birth outcomes for twins in this cohort was strong for long IPIs and weak for exposure to short IPIs.

### Supplementary Information

**Additional file 1: Supplementary Figures 1 and 2.**

**Additional file 2: Supplementary Tables 1, 2 and 3.**

## Data Availability

The data that support the findings of this study are available from are owned by the government departments, but restrictions apply to the availability of these data, which were used under license for the current study, and so are not publicly available. The current Human Research Ethics Committee approvals were obtained for public sharing and presentation of data on group level only, meaning the data used in this study cannot be shared by the authors. Collaborative research may be conducted according to the ethical requirements and relevant privacy legislations. Potential collaborators should contact author GP with their expression of interest. The steps involved in seeking permission for linkage and use of the data used in this study are the same for all researchers.

## Abbreviations

AUSEI06: Australian Socioeconomic Index 2006
CI: Confidence Interval
IPI: Interpregnancy Interval
LBW: Low Birth Weight
OR: Odds ratio
RoR: Ratio of Risk ratios
SGA: Small for Gestational Age
WA: Western Australia
