# Supplementary material for: Interpregnancy interval and adverse birth outcomes: a population-based cohort study of twins
Source: BMC Pregnancy Childbirth. 2024 Jan 31;24:96. doi: 10.1186/s12884-023-06119-x (PMC10832241; doi:10.1186/s12884-023-06119-x)
Supplement: Supplementary file 1 — Additional file 1: Supplementary Figures 1 and 2. [file 12884_2023_6119_MOESM1_ESM.docx]

**Supplementary Figure 1.** Model of Interpregnancy Interval (IPI) Cohort and the Post-Birth IPI Cohort.


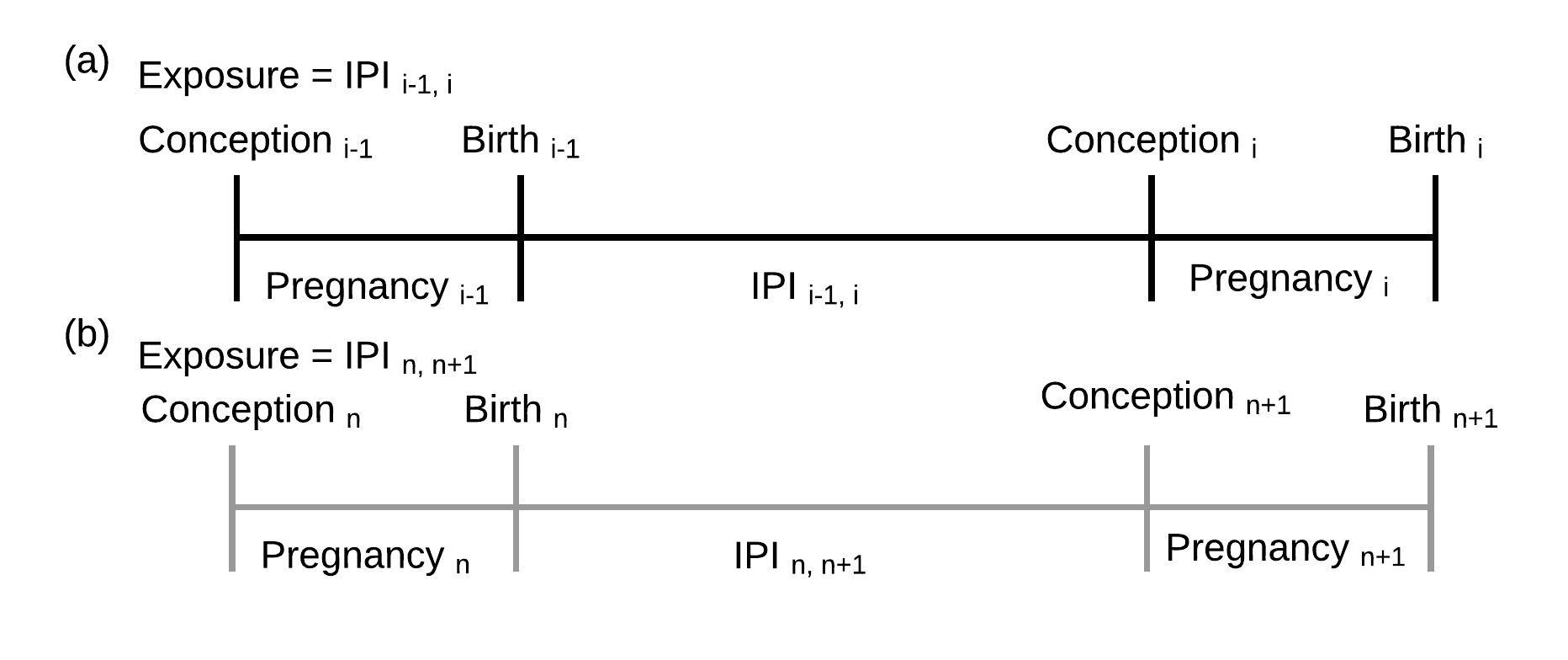


(a) IPI cohort; Conception_i_, Birth_i_, and Pregnancy_i_ represent the index twin pregnancy for the IPI cohort. Conception_i-1_, Birth_i-1_, and Pregnancy_i -1_ represent the immediately prior pregnancy to the index pregnancy for the IPI cohort, regardless of the pregnancy type (singleton or multifetal pregnancy). IPI _i-1,i_ refers to the IPI between the index twin pregnancy and the immediately prior pregnancy. (b) Post-birth IPI cohort; Conception_n_, Birth_n_, and Pregnancy_n_ represent the index twin pregnancy for the post-birth IPI cohort. Conception_n+1_, Birth_n+1_, and Pregnancy_n+1_ represent the immediately subsequent pregnancy, regardless of the pregnancy type (singleton or multifetal pregnancy). IPI_n,n+1_ refers to the IPI between the index twin pregnancy and the immediately subsequent pregnancy.

**Supplementary Figure 2.** Directed Acyclic Graph representing the associations between interpregnancy intervals, post-birth interpregnancy intervals and adverse birth outcomes in twin pregnancies.
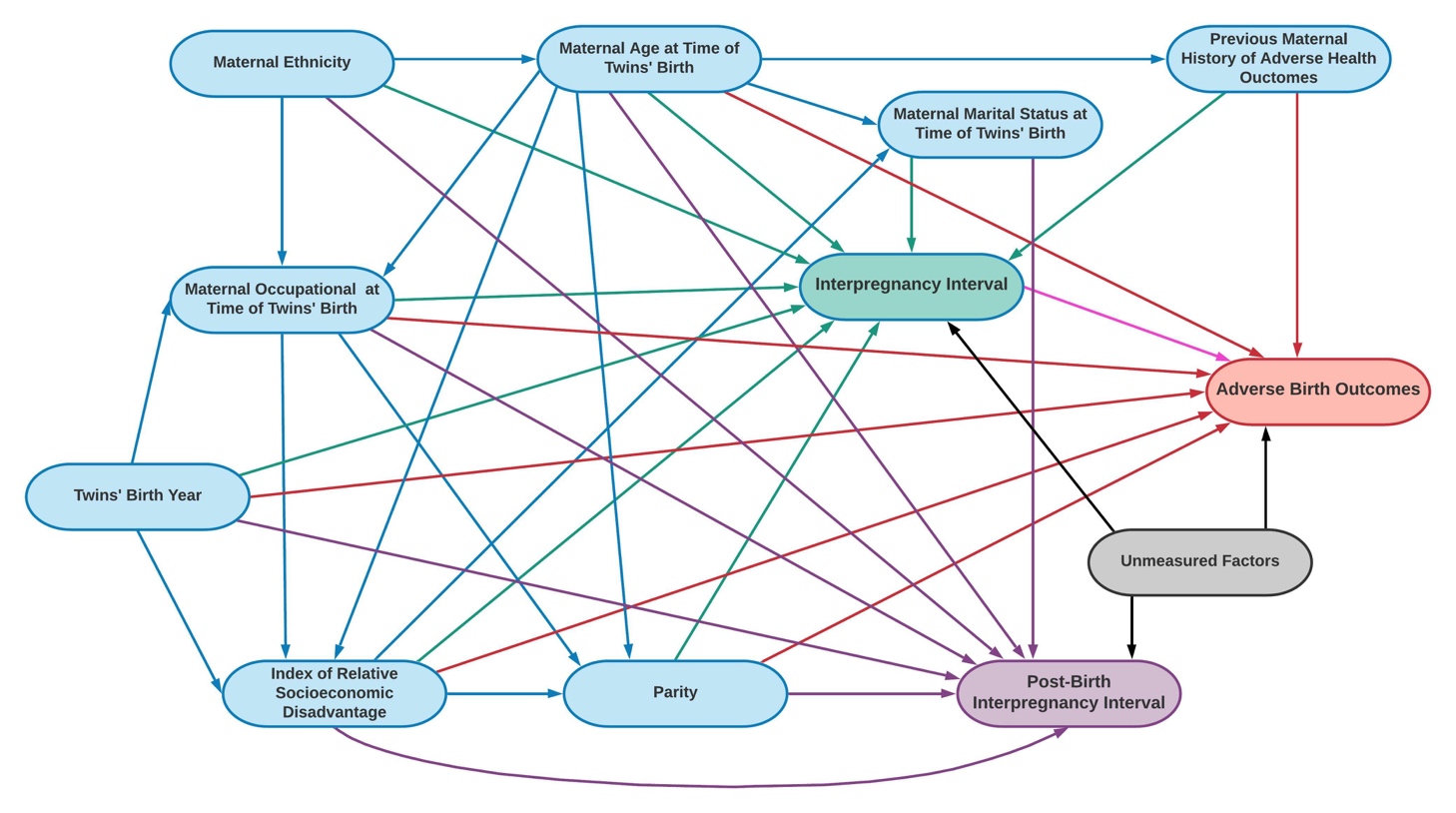


Exposures, outcomes, adjustment variables, and unmeasured factors are denoted in green, red, blue, and grey respectively. Unmeasured and unknown covariates are denoted in grey and negative control exposure is denoted in purple. Exposure: Interpregnancy Interval (IPI) was defined as the time between the birth of twins (i.e., IPI cohort pregnancy) and the start of the subsequent pregnancy. Outcome measure: adverse birth outcomes in twin pregnancy.

Negative control: Post-birth IPI was defined as the time between the birth of the twins (i.e., post-birth IPI cohort pregnancy) and the start of pregnancy of the immediately subsequent pregnancy and the relationship between post-birth IPI and the risk of adverse birth outcomes in the previous pregnancy was assessed.
