# Supplementary material for: Interpregnancy interval and adverse birth outcomes: a population-based cohort study of twins
Source: BMC Pregnancy Childbirth. 2024 Jan 31;24:96. doi: 10.1186/s12884-023-06119-x (PMC10832241; doi:10.1186/s12884-023-06119-x)
Supplement: Supplementary file 2 — Additional file 2: Supplementary Tables 1, 2 and 3. [file 12884_2023_6119_MOESM2_ESM.docx]

**Supplementary Table 1.** Comparison of results from the complete case dataset (*n*=5,438) versus imputed dataset (*n*=6,336) to estimate the adjusted Risk Ratio (RR)^1^ between; preterm birth, early preterm birth, and at least one twin being classified as low birth weight or small for gestational age, and interpregnancy intervals in twin pregnancies.

| **Outcome Variable and Cohort** | | **Interpregnancy Interval** (months) | | | | | | |
| --- | --- | --- | --- | --- | --- | --- | --- | --- |
|  |  | aRR [95% CI]^1^ | | | | | | |
|  |  | <6 | 6-11 | 12-17 | 18-23 | 24-59 | 60-119 | ≥120 |
| **Preterm Birth** | **Complete Cases**  *n=*2,927 (53.8)^2^ | 1.05 [0.93-1.18] | 0.96 [0.87-1.06] | 0.97 [0.89-1.06] | 1 [referent] | 1.02 [0.95-1.1] | **1.10 [1.01-1.21]^*^** | **1.24 [1.09-1.41]^***^** |
|  | **Imputed Cases**^3^  *n=*3,353 (52.9) | 1.05 [0.94-1.18] | 0.99 [0.90-1.08] | 0.97 [0.89-1.05] | 1 [referent] | 1.04 [0.97-1.12] | **1.12 [1.02-1.22]^**^** | **1.25 [1.10-1.41]^***^** |
| **Early Preterm Birth** | **Complete Cases**  *n=*830 (15.3) | **1.34 [1.00-1.79]^*^** | 1.02 [0.80-1.31] | 0.98 [0.78-1.23] | 1 [referent] | 1.1 [0.91-1.34] | 1.17 [0.92-1.5] | 1.24 [0.86-1.81] |
|  | **Imputed Cases**  *n=*964 (15.2) | **1.41 [1.08-1.83]^*^** | 1.17 [0.94-1.46] | 0.99 [0.8-1.23] | 1 [referent] | 1.16 [0.96-1.39] | 1.19 [0.94-1.51] | **1.42 [1.01-2.00]^*^** |
| **Small for Gestational Age** | **Complete Cases**  *n=*831 (15.3) | **1.39 [1.01-1.91]^*^** | **1.32 [1.04-1.69]^*^** | **1.28 [1.02-1.61]^*^** | 1 [referent] | **1.23 [1.01-1.51]^*^** | **1.29 [1.01-1.66]^*^** | 1.33 [0.90-1.99] |
|  | **Imputed Cases** *n=*983 (15.5) | 1.20 [0.90-1.59] | **1.24 [1.01-1.54]^*^** | 1.14 [0.93-1.40] | 1 [referent] | 1.14 [0.95-1.36] | 1.19 [0.95-1.51] | 1.30 [0.90-1.86] |
| **Low Birth Weight** | **Complete Cases**  *n=*3,081 (56.7) | **1.19 [1.07-1.33]^**^** | 1.07 [0.98-1.17] | 1.05 [0.96-1.14] | 1 [referent] | 1.07 [1.00-1.16] | **1.19 [1.08-1.30]^***^** | **1.19 [1.04-1.36]^*^** |
|  | **Imputed Cases** *n=*3,590 (56.7) | **1.16 [1.06-1.28]^**^** | **1.09 [1.01-1.19]^*^** | 1.01 [0.93-1.09] | 1 [referent] | 1.06 [0.99-1.14] | **1.17 [1.08-1.28]^***^** | **1.20 [1.05-1.36]^**^** |

IPI was defined as the time between the birth of twins (i.e., IPI cohort pregnancy) and the start of the subsequent pregnancy.

^1^Data is presented as relative risk [95% Confidence Intervals].

^2^Number of pregnancies (percentage of pregnancies) with each respective outcome.

^3^All data was based on pooled analysis from 20 imputed datasets for parity, birth year category, maternal ethnicity, maternal marital status at time of birth, maternal age at time of birth, maternal occupational status scale at time of birth, previous maternal history for each respective outcome variable, and IRSD category, with respect to the twin pregnancy.

^***^p<0.001, ^**^p<0.01, ^*^p<0.05.

**Supplementary Table 2.** Comparison of results from the complete case dataset (*n*=2,722) versus imputed dataset (*n*=3,531) to estimate the adjusted Risk Ratio (RR)^1^ between; preterm birth, early preterm birth, and at least one twin being classified as low birth weight or small for gestational age, and post-birth interpregnancy intervals in twin pregnancies.

| **Outcome Variable and Cohort** | | **Post-Birth Interpregnancy Interval** (months) | | | | | | |
| --- | --- | --- | --- | --- | --- | --- | --- | --- |
|  |  | aRR [95% CI]^1^ | | | | | | |
|  |  | <6 | 6-11 | 12-17 | 18-23 | 24-59 | 60-119 | ≥120 |
| **Preterm Birth** | **Complete Cases**  *n=*1,668 (61.3)^2^ | **1.33 [1.18-1.51]^***^** | **1.23 [1.10-1.38]^***^** | 1.04 [0.92-1.17] | 1 [referent] | 0.99 [0.89-1.01] | 0.94 [0.82-1.07] | 0.93 [0.73-1.18] |
|  | **Imputed Cases**^3^  *n=*2,112 (59.8) | **1.37 [1.22-1.53]^***^** | **1.22 [1.10-1.36]^***^** | 1.08 [0.97-1.20] | 1 [referent] | 1.01 [0.92-1.11] | 0.96 [0.85-1.08] | 0.98 [0.80-1.21] |
| **Early Preterm Birth** | **Complete Cases**  *n=*652 (24.0) | **1.82 [1.40-2.37]^***^** | **1.38 [1.08-1.77]^**^** | 0.97 [0.75-1.26] | 1 [referent] | 0.83 [0.66-1.03] | 0.81 [0.61-1.07] | 0.60 [0.33-1.09] |
|  | **Imputed Cases** *n=*842 (23.8) | **2.03 [1.61-2.55]^***^** | **1.45 [1.16-1.81]^**^** | 0.99 [0.78-1.26] | 1 [referent] | 0.88 [0.72-1.07] | 0.84 [0.66-1.08] | 0.73 [0.45-1.19] |
| **Small for Gestational Age** | **Complete Cases**  *n=*599 (22.0) | **1.54 [1.17-2.04]^**^** | 1.04 [0.80-1.35] | 0.79 [0.60-1.03] | 1 [referent] | **0.79 [0.63-0.99]^*^** | 0.78 [0.58-1.03] | 1.04 [0.66-1.64] |
|  | **Imputed Cases** *n=*821 (23.3) | **1.50 [1.17-1.92]^**^** | 1.13 [0.90-1.42] | 0.85 [0.67-1.08] | 1 [referent] | 0.86 [0.71-1.05] | 0.90 [0.71-1.14] | 0.93 [0.61-1.41] |
| **Low Birth Weight** | **Complete Cases**  *n=*1,859 (68.3) | **1.30 [1.17-1.44]^***^** | **1.19 [1.08-1.32]^***^** | 1.02 [0.92-1.14] | 1 [referent] | 1.02 [0.94-1.12] | 1.00 [0.89-1.11] | 0.90 [0.73-1.12] |
|  | **Imputed Cases**  *n=*2,422 (68.6) | **1.30 [1.19-1.42]^***^** | **1.17 [1.07-1.27]^***^** | 1.03 [0.94-1.12] | 1 [referent] | 1.01 [0.93-1.09] | 0.99 [0.90-1.09] | 0.87 [0.72-1.05] |

Post-birth IPI was defined as the time between the birth of the twins (i.e., post-birth IPI cohort pregnancy) and the start of pregnancy of the immediately subsequent pregnancy.

^1^Data is presented as relative risk [95% Confidence Intervals].

^2^Number of pregnancies (percentage of pregnancies) with each respective outcome.

^2^All data was based on pooled analysis from 20 imputed datasets for parity, birth year category, maternal ethnicity, maternal marital status at time of birth, maternal age at time of birth, maternal occupational status scale at time of birth, previous maternal history for each respective outcome variable, and IRSD category, with respect to the twin pregnancy.

^***^p<0.001, ^**^p<0.01, ^*^p<0.05.

**Supplementary Table 3.** Comparison of results from the imputed dataset adjusted for socioeconomic status (SES) at the time of the previous pregnancy compared to the imputed dataset adjusted for SES at the time of the twin pregnancy to estimate the adjusted Risk Ratio (RR)^1^ between interpregnancy intervals and adverse birth outcomes in twin pregnancies.

| **Outcome Variable and Cohort** | | **Interpregnancy Interval** (months) | | | | | | |
| --- | --- | --- | --- | --- | --- | --- | --- | --- |
|  |  | aRR [95% CI]^1^ | | | | | | |
|  |  | <6 | 6-11 | 12-17 | 18-23 | 24-59 | 60-119 | ≥120 |
| **Preterm Birth** | **Adjusted for previous pregnancy SES variables**^2^ | 1.05 [0.94-1.17] | 0.98 [0.90-1.08] | 0.97 [0.89-1.05] | 1 [referent] | 1.04 [0.97-1.11] | **1.12 [1.02-1.22]^*^** | **1.24 [1.10-1.41]^***^** |
|  | **Imputed Cases**^3^ | 1.05 [0.94-1.18] | 0.99 [0.90-1.08] | 0.97 [0.89-1.05] | 1 [referent] | 1.04 [0.97-1.12] | **1.12 [1.02-1.22]^**^** | **1.25 [1.10-1.41]^***^** |
| **Early Preterm Birth** | **Adjusted for previous pregnancy SES variables** | **1.39 [1.07-1.81]^*^** | 1.16 [0.93-1.45] | 0.99 [0.79-1.23] | 1 [referent] | 1.15 [0.96-1.38] | 1.17 [0.92-1.48] | 1.36 [0.96-1.92] |
|  | **Imputed Cases** | **1.41 [1.08-1.83]^*^** | 1.17 [0.94-1.46] | 0.99 [0.80-1.23] | 1 [referent] | 1.16 [0.96-1.39] | 1.19 [0.94-1.51] | **1.42 [1.01-2.00]^*^** |
| **Small for Gestational Age** | **Adjusted for previous pregnancy SES variables** | 1.20 [0.90-1.59] | **1.25 [1.01-1.55]^*^** | 1.14 [0.93-1.40] | 1 [referent] | 1.13 [0.95-1.36] | 1.17 [0.93-1.48] | 1.23 [0.86-1.78] |
|  | **Imputed Cases** | 1.20 [0.90-1.59] | **1.24 [1.01-1.54]^*^** | 1.14 [0.93-1.40] | 1 [referent] | 1.14 [0.95-1.36] | 1.19 [0.95-1.51] | 1.30 [0.90-1.86] |
| **Low Birth Weight** | **Adjusted for previous pregnancy SES variables** | **1.17 [1.06-1.29]^**^** | **1.09 [1.01-1.18]^*^** | 1.01 [0.93-1.09] | 1 [referent] | 1.06 [0.99-1.14] | **1.17 [1.08-1.28]^***^** | **1.19 [1.05-1.35]^**^** |
|  | **Imputed Cases** | **1.16 [1.06-1.28]^**^** | **1.09 [1.01-1.19]^*^** | 1.01 [0.93-1.09] | 1 [referent] | 1.06 [0.99-1.14] | **1.17 [1.08-1.28]^***^** | **1.20 [1.05-1.36]^**^** |

IPI was defined as the time between the birth of twins (i.e., IPI cohort pregnancy) and the start of the subsequent pregnancy.

^1^Data is presented as relative risk [95% Confidence Intervals].

^2^All data was based on pooled analysis from 20 imputed datasets for parity, birth year category, maternal ethnicity, maternal marital status at time of birth, maternal age at time of birth, maternal occupational status scale at time of the previous pregnancy, previous maternal history for each respective outcome variable, and IRSD category at time of the previous pregnancy, with respect to the twin pregnancy.

^3^All data was based on pooled analysis from 20 imputed datasets for parity, birth year category, maternal ethnicity, maternal marital status at time of birth, maternal age at time of birth, maternal occupational status scale at time of birth, previous maternal history for each respective outcome variable, and IRSD category, with respect to the twin pregnancy.

^***^p<0.001, ^**^p<0.01, ^*^p<0.05.
